# Age-Friendly Communities and Aging in Place: Findings From Latent Profile Analysis

**DOI:** 10.1093/geroni/igab046.2141

**Published:** 2021-12-17

**Authors:** Seon Kim, Kyeongmo Kim, junpyo kim

**Affiliations:** 1 Virginia commonwealth university, Richmond, Virginia, United States; 2 Virginia Commonwealth University, Henrico, Virginia, United States; 3 Gyeongsang National University, Jinju-si, Kyongsang-namdo, Republic of Korea

## Abstract

Older adults prefer to live in their current home or community and ‘Aging in place’ has been shown to reduce the cost of caring for older adults and help their successful aging. Although age-friendly communities (AFC) initiatives have been helpful to aging in place, little has been known about the relationship between the types of AFC and aging in place. Using the 2017 AARP Age-Friendly Community Survey, we included 1,079 adults aged 65 or older. We measured aging in place as ‘move to a different community’, ‘move into a different residence within your current community’, and ‘stay in your current residence’, and included eight AFC constructs. We identified the type of AFC using Latent Profile Analysis: low-friendly, mid-friendly, and high-friendly. We also ran multinomial logistic regression to examine whether the types of AFC were associated with aging in place. Of the total participants, 26.0% lived in the low-friendly community, 23.7% in the mid-friendly community, and 50.3% in the high-friendly community. Older adults living in the high-friendly community were more likely to stay in the current residence (64.7%) than those in the low-friendly (47.1%) (χ2=28.680, p<.001). Also, older adults living in the low-friendly community (OR=3.05, p<.001) and the mid-friendly community (OR=1.42, p<.10) were more likely to move to a different community compared to those living in the high-friendly community. This result suggests that it is important to build an AFC to promote aging in place. For the growing number of older adults' lives, policymakers should consider expanding the AFC initiatives.

